# Association between Sexual Activity and Human Papillomavirus (HPV) Vaccine Initiation and Completion among College Students

**DOI:** 10.3390/vaccines10122079

**Published:** 2022-12-05

**Authors:** Eric Adjei Boakye, Stacey L. McKinney, Kelli D. Whittington, Valerie E. Boyer, Maria C. Franca, Minjee Lee, Richard C. McKinnies, Sandra K. Collins, Mary A. Gerend

**Affiliations:** 1 Department of Otolaryngology—Head and Neck Surgery, Henry Ford Health System, One Ford Place, Detroit, MI 48202, USA; 2 Department of Public Health Sciences, Henry Ford Health System, One Ford Place, Detroit, MI 48202, USA; 3 Department of Dental Hygiene, School of Health Sciences, Southern Illinois University, 1263 Lincoln Dr, Carbondale, IL 62901, USA; 4 Department of Nursing, School of Health Sciences, Southern Illinois University, 1263 Lincoln Dr, Carbondale, IL 62901, USA; 5 Department of Communication Disorders and Sciences, School of Health Sciences, Southern Illinois University, 1263 Lincoln Dr, Carbondale, IL 62901, USA; 6 Department of Population Science and Policy, Southern Illinois University School of Medicine, 201 E. Madison Street, Springfield, IL 62794, USA; 7 Department of Radiologic Sciences, School of Health Sciences, Southern Illinois University, 1263 Lincoln Dr, Carbondale, IL 62901, USA; 8 Department of Health Care Management, School of Health Sciences, Southern Illinois University, 1263 Lincoln Dr, Carbondale, IL 62901, USA; 9 Department of Behavioral Sciences and Social Medicine, Florida State University College of Medicine, 1115 West Call Street, Tallahassee, FL 32306, USA

**Keywords:** HPV vaccine initiation and completion, human papillomavirus, vaccine coverage, college students, sexual behavior

## Abstract

HPV vaccination is most effective if received before initiation of sexual activity. Previous studies suggested that young adult women who were not sexually active were not interested in receiving the vaccine because they did not think it was necessary. Whether this misperception is still prevalent today—and also shared by men—is unknown. This study examined whether sexual activity was associated with HPV vaccine uptake (initiation and completion) among university students. A cross-sectional study was conducted between February and May 2021 among students (*n* = 951) at a public Midwestern University. Sexual activity was categorized as “never” or “ever” had oral and/or vaginal sex. Outcome variables were HPV vaccine initiation, defined as receipt of ≥1 dose, and completion, defined as receipt of ≥3 doses. Multivariable logistic regression models estimated the association between sexual activity and HPV vaccine uptake, adjusting for sociodemographic factors. Approximately 18% of students reported never engaging in sexual activity. Overall, 45.5% initiated the HPV vaccine, and 16.5% completed the vaccine series. After adjusting for covariates, compared to students that reported never engaging in sexual activity, those that had ever engaged in sexual activity were more likely to have initiated the vaccine series (aOR = 2.06, 95% CI: 1.34–3.17); however, no difference was observed for completion. HPV vaccination was low; sexually naïve students were less likely to initiate the HPV vaccine. Since sexually naïve students may benefit from receiving the HPV vaccination, targeted interventions should be implemented towards this population to help increase vaccination rates and prevent HPV-associated diseases.

## 1. Introduction

Human papillomavirus (HPV) is the most common sexually transmitted infection in the United States [1] with an estimated 14 million new cases of HPV infection each year [2]. There are over 100 types of HPV [3]. Although most subtypes are cleared by the body without any harm [4], persistent HPV infections can lead to genital warts, precancers, anogenital cancers, oropharyngeal cancers, and second primary cancers [1,5,6,7,8]. In the United States, HPV accounts for about 44,000 HPV-associated cancers per year: about 25,000 among women, and about 19,000 among men [9]. The incidence of HPV infection is highest among individuals in their late teens and early twenties. Individuals in this age group are more likely to engage in risky sexual behaviors such as having multiple sexual partners and failing to use protection against sexually transmitted infections [2]. Campus-level studies provide support that sexual risk-taking behaviors are pervasive among university students [10,11,12]. It is estimated that 74% of HPV infections occur among men and women aged 15–24 [13]. As such, university students, who are primarily in the 18- to 24-year age group, are concentrated in this increased risk age group. Given that many young adults who have not been exposed to HPV can still greatly benefit from the HPV vaccine, there needs to be a better understanding of the factors affecting HPV vaccination rates among college students.

It is estimated that the HPV vaccine could prevent over 90% of HPV-attributable cancers [14]. The Advisory Committee on Immunization Practices (ACIP) recommends routine HPV vaccination for adolescents between 11 and 12 years of age, although the vaccine can be given to children as young as age 9 [15,16]. Catch-up vaccination is recommended for both males and females aged 13–26 years who did not receive the vaccine when they were younger. Although adults aged 27–45 can receive the HPV vaccine, the public health benefit of vaccinating individuals in this age range is relatively small [15,16]. In the Fall of 2016, the ACIP stated that adolescents who initiated the vaccine at 9–14 years of age need only two doses received 6 months apart [17]. However, those who initiated the vaccine at the age of 15 years or older still need the three doses. The HPV vaccine is safe and effective; however, vaccine uptake remains lower than desired. Rates have risen in the adolescent population in the United States. In 2020, 75.1% of adolescents aged 13–17 years had received ≥1 dose of HPV vaccine (77.1% for girls and 73.1% for boys) and 58.6% had completed the series (61.4% for girls and 56.0% for boys) [18]. For young adults, the traditional college-age demographic of 18–26 years, uptake is lower with initiation estimated at 39.9% and completion estimated at 21.5% as of 2018 [19].

The primary risk factors for acquiring HPV are generally associated with sexual activity [20,21]. The vaccine is most effective if received before initiation of sexual activity [22]. Previous literature has examined association between sexual behavior and HPV vaccination among university-aged women [23,24,25]. They found that young women who were not sexually active were not interested in receiving the vaccine because they did not think it was necessary [23,24,25]. Studies are needed to access whether this misperception persists 15 years later, and also to include young men. In addition, although young adults are at the greatest risk of HPV infection, extensive vaccine promotion and intervention efforts directed toward 11–12-year-olds has led to the near exclusion of strategies targeting young adults. The university environment represents an optimal scenario to better understand the needs of these young adults affected by this public health concern and to provide interventions to increase HPV vaccination, as college students can legally consent to receiving HPV vaccination on campus. This study examined if sexual activity was associated with HPV vaccination uptake (initiation and completion) among university students. We hypothesized that vaccination uptake will be higher among sexually active students compared to sexually naïve students.

## 2. Materials and Methods

### 2.1. Study Population and Recruitment

We conducted a cross-sectional study among college students enrolled at a public Midwestern university. The school enrolls students from all 50 states as well as more than 100 countries. The questionnaire was administered using SurveyMonkey, it was distributed online to randomly selected enrolled students between February and May 2021. The questionnaire elicited sociodemographic information, sexual behavior (ever had oral/vaginal sex, number of oral/vaginal sexual partners, age of oral/vaginal sexual debut), as well as questions regarding HPV vaccination history (received HPV vaccination, how many doses received, intent to receive vaccination if not vaccinated already, barriers to receiving the vaccine). Participants were informed of the anonymous nature of the survey and the objective of the study, and informed consent was obtained. The introductory email included a link to the questionnaire (SurveyMonkey). Responses to the questionnaire were recorded anonymously. Participation was noncompulsory and there were no incentives offered for participation. We excluded students who were over 38 years old. The survey was distributed online to 3142 randomly selected students and 1089 completed the survey for a response rate of 34.7%. After limiting the data to students who were between 18 and 38 years (who constituted over 95% of the student body), a total of 951 students (621 females and 330 males) were included in the study. The upper age was selected because students who were between the ages of 18 and 26 years in 2021 would have been eligible to have received the HPV vaccine, based on the year of licensure in the US. The study was approved by the Southern Illinois University Institutional Review Board.

### 2.2. Measures

The main outcome variable was HPV vaccination uptake. HPV vaccine initiation was assessed with the question “*Have you ever received an HPV shot or vaccine? The vaccines are sometimes called CERVARIX or GARDASIL*”. Participants who responded yes were subsequently asked how many doses of the HPV vaccine they had received. Participants who had received ≥1 dose were deemed to have initiated the vaccination series, while participants with ≥3 vaccine doses were deemed to have completed the series. The secondary outcome was willingness to receive the vaccine. Participants who had not received the vaccine were asked how likely they were to receive the vaccine if offered. For regression analysis, we grouped responses extremely likely and very likely as “willing to receive the vaccine” and extremely unlikely, very unlikely, and somewhat likely as “unwilling to receive the vaccine”.

The main independent variable was sexual activity assessed with these questions: *“Have you ever had vaginal sexual intercourse?”, and “Have you ever had oral sex*?” Responses were categorized as “never” for those that responded no oral and no vaginal sex, or “ever” for those that had ever had oral and/or vaginal sex. To further examine the effects of sexual behavior and HPV vaccination, we examined the number of sexual partners and age of sexual debut among students who were sexually active. Number of sexual partners was categorized as “1–3” or “≥4” and age of sexual debut as “≤15 years”, “16–17 years”, or “≥18 years”. We also assessed frequency of using protection during vaginal sex into one of three categories (all the time, sometimes/often, almost never/never).

Covariates included age, gender (women, men), race/ethnicity (non-Hispanic [NH] white, NH black, Hispanic, NH other), relationship status (married, single but dating, single and not dating), country of birth (USA, non-USA), residential area before college (urban [codes 1–3], rural [codes 4–9] based on rural–urban continuum codes), and year of study (first year, second year, third year, fourth year, graduate student).

### 2.3. Statistical Analysis

Descriptive statistics were used to analyze characteristics of the study participants. Sociodemographic characteristics and sexual behavioral risk factors were compared by sexual activity using chi-square tests for categorical variables and independent samples t-test for continuous variables. Next, two multivariable logistic regression models were used to examine the association between HPV vaccine uptake and sexual activity. Adjustments for age, sex, race/ethnicity, relationship status, country of birth, academic level, and rural-urban status were made. In the subgroup of sexually active students (*n* = 795), multivariable logistic regression models estimated the association between vaccine uptake, and number of sexual partners and age of sexual debut adjusting for the covariates listed above. In the subgroup of unvaccinated students (*n* = 518), a multivariable logistic regression examined the association between willingness to get vaccinated, and age, sex, race/ethnicity, relationship status, country of birth, academic level, rural-urban status, and sexual activity. Adjusted odds ratios with their corresponding 95% confidence intervals were reported. Analyses were performed using SAS statistical software version 9.4 (SAS Institute Inc, Cary, NC, USA). All statistical analyses were 2-tailed, and the significance level was set at *p* < 0.05.

## 3. Results

Table 1 summarizes the characteristics of the students who participated in the study, overall and by sexual naivety. A total of 951 students (433 of whom had received at least one dose of the vaccine and 581 of whom were unvaccinated) were included in the study, of whom 156 (18.1%) reported they had never engaged in sexual activity (oral and/or vaginal sex). The average age of the study population was 22.4 years. The majority were women (65.3%), non-Hispanic white (71.6%), were born in the USA (89.4%), and resided in an urban area (75.3%). When stratified by sexual activity, compared to students who had ever engaged in sexual activity, those that had not were younger (20.8 vs. 22.7; *p* < 0.0001), single and not dating (82.7% vs. 37.9%; *p* < 0.0001), and were freshman/sophomores (51.3 vs. 28.5; *p* < 0.0001).

**Figure 1:**
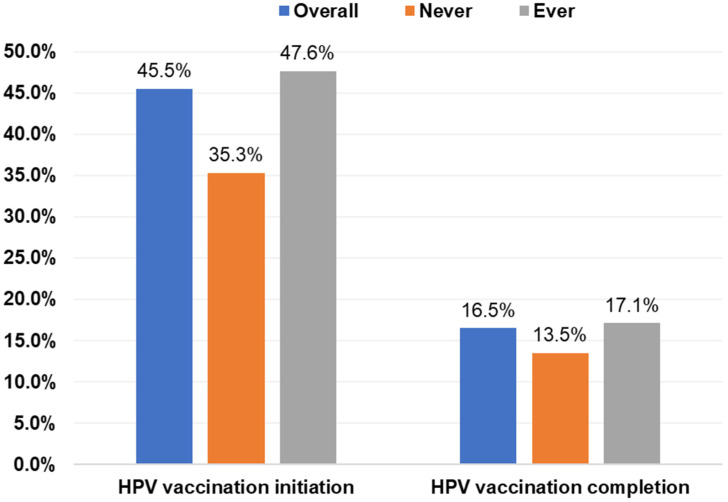
HPV vaccine initiation and completion rates of college students by sexual activity. Note: There were significant differences between sexual activity and vaccine initiation (*p* = 0.0048) but not completion (*p* = 0.2622) on the basis of chi-square tests. HPV, human papillomavirus.

**Figure 2:**
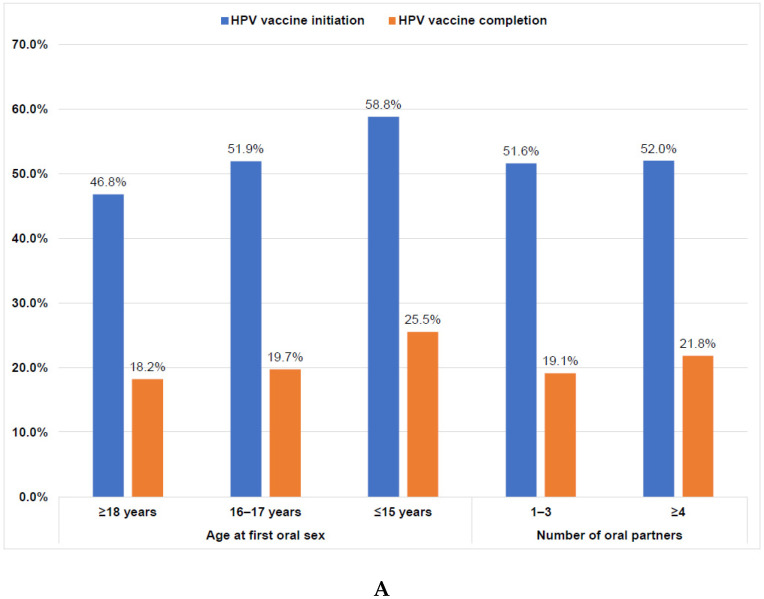

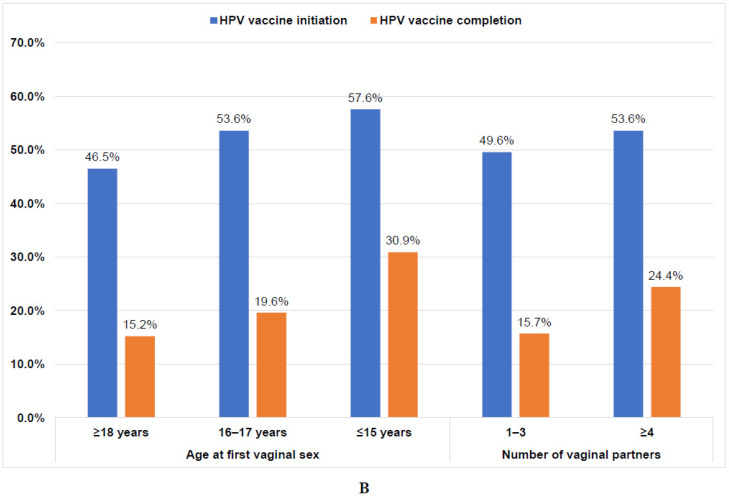
(**A**). HPV vaccine initiation and completion rates of sexually active college students by age of first oral sex and number of lifetime oral partners (*n* = 795). Note: There were no significant differences between sexual activity and vaccine initiation (*p* = 0.2068) or completion (*p* = 0.4069) on the basis of chi-square tests. HPV, human papillomavirus. (**B**). HPV vaccine initiation and completion rates of sexually active college students by age of first vaginal sex and number of lifetime vaginal partners (*n* = 795). Note: There were significant differences between sexual activity and vaccine initiation (*p* = 0.0011) and completion (*p* = 0.0078) on the basis of chi-square tests. HPV, human papillomavirus.

Overall, 45.5% had initiated the HPV vaccine, and 16.5% had completed the vaccination series (Figure 1). Initiation was higher among students who had ever engaged in sexual activity (47.6%) than those who were sexually naïve (35.3%; *p* = 0.0048); however, completion rates were similar (Figure 1). Among students who had ever engaged in sexual activity, those who had their oral sexual debut at ≤15 years had the highest initiation rate (58.8%) while those who had oral sexual debut at ≥18 years had the lowest initiation rate (46.8%; *p* = 0.0739). Completion rates were similar by age of oral sexual debut (Figure 2A). Initiation and completion rates were also similar for students with 1–3 or ≥4 oral sexual partners (Figure 2A). Students who had vaginal sexual debut at ≤15 years had the highest initiation rate (57.6%) and completion rates (30.9%), while those who had vaginal sexual debut at ≥18 years had the lowest rates (46.5% and 15.2%, respectively; *p* = 0.0011; Figure 2B). Initiation rates were similar for students with 1–3 or ≥4 vaginal sexual partners, but completion rates were higher for those with ≥4 vaginal sexual partners (24.4%) than those with 1–3 partners (15.7%; *p* = 0.0078; Figure 2B).

Regression results of the association between sexual activity and HPV vaccine uptake are presented in Table 2. After adjusting for covariates, compared to students who reported never engaging in sexual activity, those that had ever engaged in sexual activity were more likely to have initiated (aOR = 2.06, 95% CI: 1.34–3.17) the HPV vaccine; however, no difference was observed for completion. Lower odds of HPV vaccine initiation were associated with younger age (aOR = 0.89, 95% CI: 0.84–0.95). Compared to female students, male students were less likely to have initiated the vaccine series (aOR = 0.32, 95% CI: 0.23–0.45), as well students who resided in rural areas (aOR = 0.67, 95% CI: 0.47–0.95) compared to those that resided in urban areas. Similarly, freshman/sophomore (aOR = 0.54, 95% CI: 0.31–0.95) or junior/senior (aOR = 0.63, 95% CI: 0.41–0.99) students were less likely to have initiated the vaccine series. The only variable associated with vaccination completion was gender, such that male students were 75% less likely to have completed the series compared to female students.

Table 3 presents the results of the models assessing the association between HPV vaccination and number of sexual partners (Model 1) and age of sexual debut (Model 2) among sexually active college students. Students who have had ≥4 vaginal sexual partners (aOR = 2.18, 95% CI: 1.18–4.05) were more likely to complete the vaccine series compared to those with 1–3 partners, however, there was no difference for initiation. No significant difference was found for number of oral sexual partners. Students who had their vaginal sexual debut under age 18 were more likely to complete the vaccine series than those who had their sexual debut ≥18 years old. No relationship was observed for oral sexual debut and vaccine uptake. Further, among unvaccinated students, there was no association between sexual activity and willingness to receive the vaccination (Table 4).

## 4. Discussion

This study assessed the association between HPV vaccine initiation and completion and history of sexual activity among college students at a public Midwestern University. A little less than half (45.5%) had initiated the HPV vaccine, while less than one fifth (16.5%) completed the vaccination series. These initiation and completion results are similar to findings from other studies involving college students or 18–26-year-olds [19,26,27,28]. Further, results revealed that students who are sexually naïve are less likely to initiate the HPV vaccine compared to those who had ever engaged in sexual activity. Male students and rural residents were less likely to initiate the vaccine than female and urban students. Among students who had ever engaged in sexual activity, HPV initiation and completion were similar for number of oral sexual partners and age at oral sexual debut but higher among those with ≥4 vaginal sexual partners and those had vaginal sexual debut at ≥18 years. Among unvaccinated students, more than half (56%) indicated they are willing to get the vaccine if it was recommended. Therefore, healthcare professionals, especially school nurses and providers, should use every encounter with a student as an opportunity to recommend the HPV vaccine. Increasing college students’ understanding of the severity of the virus and promoting preventive health measures can lead to individuals self-awareness of sexual behaviors and decrease adverse health outcomes [28]. This might help increase vaccine uptake and thus decrease HPV-associated diseases.

We found that students who had never engaged in any sexual activity were less likely to initiate the HPV vaccine compared to those who had ever engaged in sexual activity. Our finding is consistent with previous studies conducted over a decade ago and found that students who are not sexually active see less need for the vaccine [23,24]. The fact that we are still seeing lower rates of uptake among sexually naïve participants suggests this belief is persisting and healthcare professionals and the research community have not done a good job emphasizing the benefits of the vaccine for these individuals. The low vaccination among sexually naïve students creates an important opportunity for intervention since this subgroup might benefit the most from receiving the vaccine. The ideal time for individuals to be vaccinated against HPV is prior to the onset of sexual debut [22], thus sexually naïve students might benefit more than those who have previously engaged in sexual activity.

Given the general lack of awareness and knowledge about HPV vaccination and HPV-associated cancers [29,30,31], programs to increase student knowledge about the benefits of HPV vaccination are needed. The HPV vaccine (with an average cost of $300) is free for individuals with health insurance and Vaccines for Children (VFC) program provides free vaccines for those that may not have insurance. College students in the US have health insurance as a requirement for admissions, so this should not be a barrier. Aiding in the decision-making process, healthcare providers can increase interest in HPV vaccination by avoiding describing the HPV vaccination as “optional”, and differentiating it from other adolescent vaccines, or simply failing to support it [32]. Several factors, such as limited visits to healthcare providers, and incorrect beliefs about vaccines and vaccine safety can all impact HPV vaccination acceptance. Healthcare providers, especially student health nurses and physicians and allied health staff, should use every interaction with college students to educate them about the cancer-prevention benefits of HPV vaccination and recommend it if necessary. Since college students are uniquely situated to make decisions about their health, interventions should focus on this population, especially the sexually naïve students.

Students who lived in rural areas before college were less likely to have initiated the HPV vaccination series than their urban counterparts. This finding is similar to findings from Swiecki-Sikora et al. and Adjei Boakye et al. who reported lower HPV vaccination rates among adolescents in rural communities compared to urban communities [33,34]. Lower vaccination in rural areas could be due to several reasons. Rural residents face many challenges such as travel distance, transportation complications, inability to take time off work, and difficulty accessing preventive health care [33,35]. Since the vaccine is recommended in two or three doses, individuals may start the series but have difficulty returning to the clinic for a second/third dose. Rural residents are often characterized by poor health outcomes including lower childhood and adult immunization rates [36,37]. Additionally, individuals in rural areas generally have lower incomes [38], less education [39] and are more likely to be uninsured [33]. Limited healthcare providers may also negatively affect the availability of preventive services [33]. Rural individuals are also less aware of HPV associated cancers and HPV vaccination recommendations [40]. Finally, as HPV is a sexually transmitted infection, differences in attitudes about sexual activity between rural and urban parents could explain differences in vaccination, since rural populations tend to be more conservative. As college students from rural communities have access to HPV vaccination through campus health centers, interventions should be implemented to encourage and provide these students with the vaccine. Continued educational outreach and provider recommendation for the HPV vaccine to students should therefore remain a crucial part of increasing vaccination rates in this population.

This study has several limitations. First, although findings are consistent with prior studies among college students [23,24,25], students in the current study were recruited from one public university in the Midwestern US, and therefore the findings may not be generalizable to the entire US student population. This means our findings from college students at a different university in a different location may be different than what we found. Therefore, readers should not extrapolate our findings to all college students in the US. Second, social desirability bias may have affected students’ responses, especially on the sexual activity questions. However, the use of an anonymous survey should have reduced any such bias. Third, we relied on self-reported HPV vaccination and therefore it is possible that some students might have received the vaccination as children and did not remember or know whether they had been vaccinated. Fourth, the use of a cross-sectional survey design limits our ability to draw causal conclusions. Fifth, there was no information on age of vaccination due to recall bias among those that might have received the vaccination at an earlier age. We also did not have information on HPV infection due to the fact that most people do not get tested for HPV.

## 5. Conclusions

In the current college population, rates of both HPV vaccine initiation and completion were low. Sexual activity was an independent predictor of HPV vaccine initiation, where sexually naïve students were less likely to receive the vaccine. Further, college students from rural areas and males were less likely to receive the vaccine. Increasing HPV vaccination is the only primary preventative measure for lowering HPV infection rates, especially in the high-risk college student population. Uptake of the HPV vaccination series should be encouraged on college campuses as it will decrease rates of HPV infection and HPV-associated cancers. Future work should focus on finding the most optimal ways to increase HPV vaccination among college students. Engaging college students in the development of such interventions will help identify effective strategies that can be tailored to their needs. Future research should solicit students’ input regarding intervention content, distribution channels, and components that resonate with the target population.

## Figures and Tables

**Table 1:** Characteristics of college students, Overall and Stratified by sexual activity (*n* = 951).

	Frequency (%) or Mean ± Standard Deviation			*p*-Value
	Overall	Sexual Activity		
		Never	Ever (Oral and/or Vaginal)	
**Sexual activity**				
Never	156 (18.1)			
Ever (oral and/or vaginal)	795 (81.9)			
**Age**	22.4 ± 4.0	20.8 ± 2.7	22.7 ± 4.1	<0.0001
**Gender**				0.7312
Female	621 (65.3)	100 (64.1)	521 (65.5)	
Male	330 (34.7)	56 (35.9)	274 (34.5)	
**Race/ethnicity**				0.1765
Non-Hispanic white	681 (71.6)	115 (73.7)	566 (71.2)	
Non-Hispanic black	78 (8.2)	12 (7.7)	66 (8.2)	
Hispanic	89 (9.4)	8 (5.1)	81 (10.2)	
Non-Hispanic other	103 (10.8)	21 (13.5)	82 (10.3)	
**Relationship status**				<0.0001
Married	156 (16.4)	1 (0.6)	155 (19.5)	
Single but dating	365 (38.4)	26 (16.7)	339 (42.6)	
Single and not dating	430 (45.2)	129 (82.7)	301 (37.9)	
**Country of birth**				0.6840
USA	850 (89.4)	138 (88.5)	712 (89.6)	
Non-USA	101 (10.6)	18 (11.5)	83 (10.4)	
**Academic level**				<0.0001
Graduate student	269 (28.3)	22 (14.1)	247 (31.1)	
Junior/Senior	375 (39.4)	54 (34.6)	321 (40.4)	
Freshman/Sophomore	307 (32.3)	80 (51.3)	227 (28.5)	
**Rural-Urban status**				0.2486
Urban	661 (75.3)	100 (71.4)	561 (76.0)	
Rural	217 (24.7)	40 (28.6)	177 (24.0)	
**First generation college student**				0.0777
No	635 (67.5)	114 (73.5)	521 (66.3)	
Yes	306 (35.5)	41 (26.5)	265 (33.7)	
**Ever had vaginal sex**				
No	231 (26.1)			
Yes	653 (73.9)			
**Age at first vaginal sex**				
No vaginal sex	231 (26.3)			
≥18 years	262 (30.1)			
16–17 years	239 (27.5)			
≤15 years	140 (16.1)			
**Number of vaginal partners**				
None	231 (26.5)			
1–3	298 (34.3)			
≥4	341 (39.2)			
**Times used protection during vaginal sex**				
No vaginal sex	231 (26.5)			
All the time	187 (21.5)			
Sometime/often	299 (32.3)			
Almost never/never	154 (17.7)			
**Ever had oral sex**				
No	179 (20.7)			
Yes	685 (79.3)			
**Age at first oral sex**				
No oral sex	179 (20.8)			
≥18 years	260 (30.2)			
16–17 years	256 (29.7)			
≤15 years	167 (19.4)			
**Number of oral partners**				
None	179 (20.8)			
1–3	360 (41.8)			
≥4	322 (37.4)			

**Table 2:** Multivariable logistic regression models estimating associations between sexual activity and HPV vaccination uptake among college students, (*n* = 951).

	aOR (95% Confidence Interval)	
	HPV Vaccination Initiation	HPV Vaccination Completion
**Age**	**0.89 (0.84, 0.95)**	0.95 (0.88, 1.02)
**Gender**		
Female	Ref	Ref
Male	**0.32 (0.23, 0.45)**	**0.25 (0.15, 0.42)**
**Race/ethnicity**		
Non-Hispanic white	Ref	Ref
Non-Hispanic black	0.87 (0.49, 1.55)	1.17 (0.61, 2.27)
Hispanic	0.66 (0.39, 1.11)	0.65 (0.32, 1.31)
Non-Hispanic other	0.68 (0.38, 1.23)	0.79 (0.36, 1.75)
**Relationship status**		
Married	Ref	Ref
Single but dating	1.07 (0.68, 1.68)	0.92 (0.52, 1.62)
Single and not dating	1.17 (0.73, 1.89)	1.18 (0.66, 2.12)
**Country of birth**		
USA	Ref	Ref
Non-USA	0.54 (0.27, 1.05)	0.53 (0.21, 1.36)
**Academic level**		
Graduate student	Ref	Ref
Junior/Senior	**0.63 (0.41, 0.99)**	0.88 (0.52, 1.51)
Freshman/Sophomore	**0.54 (0.31, 0.95)**	0.64 (0.32, 1.26)
**Rural-Urban status**		
Urban	Ref	Ref
Rural	**0.67 (0.47, 0.95)**	0.76 (0.49, 1.19)
**Sexual activity**		
Never	Ref	Ref
Ever (oral and/or vaginal)	**2.06 (1.34, 3.17)**	1.46 (0.83, 2.55)

**Table 3:** Associations between lifetime number of sexual partners and age of sexual debut, and HPV vaccination uptake among sexually active college students, (*n* = 795).

	aOR (95% Confidence Interval)			
	Model 1 (# of Sexual Partners)		Model 2 (Age of Sexual Debut)	
	HPV Vaccination Initiation	HPV Vaccination Completion	HPV Vaccination Initiation	HPV Vaccination Completion
**Age**	**0.90 (0.84, 0.95)**	0.95 (0.88, 1.03)	**0.90 (0.85, 0.96)**	0.96 (0.89, 1.04)
**Gender**				
Female Male	Ref **0.35 (0.23, 0.51)**	Ref **0.22 (0.12, 0.41)**	Ref **0.34 (0.23, 0.50)**	Ref **0.22 (0.11, 0.41)**
**Race/ethnicity**				
Non-Hispanic white Non-Hispanic black Hispanic Non-Hispanic other	Ref 0.95 (0.49, 1.85) 0.47 (0.26, 0.87) 0.74 (0.36, 1.53)	Ref 1.51 (0.73, 3.13) 0.63 (0.28, 1.44) 0.90 (0.36, 2.24)	Ref 0.97 (0.50, 1.87) 0.46 (0.25, 0.84) 0.77 (0.37, 1.60)	Ref 1.53 (0.73, 3.12) 0.62 (0.27, 1.41) 1.00 (0.39, 2.46)
**Relationship status**				
Married Single but dating Single and not dating	Ref 1.13 (0.70, 1.82) 1.13 (0.68, 1.89)	Ref 0.99 (0.56, 1.78) 1.00 (0.54, 1.78)	Ref 1.15 (0.71, 1.85) 1.19 (0.71, 2.00)	Ref 1.05 (0.59, 1.89) 1.17 (0.62, 2.20)
**Country of birth**				
USA Non-USA	Ref 0.82 (0.36, 1.89)	Ref 0.76 (0.26, 2.18)	Ref 0.83 (0.36, 1.92)	Ref 0.67 (0.23, 2.00)
**Academic level**				
Graduate student Junior/Senior Freshman/Sophomore	Ref 0.67 (0.42, 1.09) 0.54 (0.29, 1.00)	Ref 0.91 (0.51, 1.63) 0.71 (0.34, 1.50)	Ref 0.69 (0.43, 1.11) 0.52 (0.29, 0.97)	Ref 0.92 (0.51, 1.65) 0.66 (0.31, 1.40)
**Rural-Urban status**				
Urban Rural	Ref 0.73 (0.48, 1.11)	Ref 0.85 (0.51, 1.42)	Ref 0.74 (0.49, 1.13)	Ref 0.89 (0.53, 1.48)
**Number of vaginal partners**				
1–3 ≥4	Ref 1.46 (0.87, 2.46)	Ref **2.18 (1.18, 4.05)**		
**Number of oral partners**				
1–3 ≥4	Ref 0.85 (0.51, 1.43)	Ref 0.73 (0.40, 1.34)		
**Age at first vaginal sex**				
≥18 years 16–17 years ≤15 years			Ref 1.25 (0.73, 2.14) 1.23 (0.63, 2.39)	Ref **2.15 (1.07, 4.33)** **3.59 (1.61, 8.02)**
**Age at first oral sex**				
≥18 years 16–17 years ≤15 years			Ref 0.94 (0.55, 1.60) 1.23 (0.63, 2.39)	Ref 0.64 (0.32, 1.26) 0.62 (0.28, 1.39)

**Table 4:** Factors associated with willingness to receive vaccination among unvaccinated college students (*n* = 518).

	Adjusted OR (95% Confidence Interval)
**Age**	**1.14 (1.06, 1.22)**
**Gender**	
Female Male	Ref 0.99 (0.65, 1.52)
**Race/ethnicity**	
Non-Hispanic white Non-Hispanic black Hispanic Non-Hispanic other	Ref 1.08 (0.47, 2.51) 0.98 (0.49, 1.96) 0.93 (0.43, 2.01)
**Relationship status**	
Married Single but dating Single and not dating	Ref 1.21 (0.63, 2.31) 0.89 (0.45, 1.74)
**Country of birth**	
USA Non-USA	Ref 1.29 (0.56, 2.94)
**Academic level**	
Graduate student Junior/Senior Freshman/Sophomore	Ref **2.29 (1.23, 4.26)** 1.52 (0.70, 3.28)
**Rural-Urban status**	
Urban Rural	Ref 0.74 (0.45, 1.19)
**Sexual activity**	
Never Ever (oral and/or vaginal)	Ref 1.18 (0.68, 2.05)

## Data Availability

Analytic data will be provided by corresponding author upon request.
